# Quantification of the in vivo stiffness and natural length of the human plantar aponeurosis during quiet standing using ultrasound elastography

**DOI:** 10.1038/s41598-022-20211-w

**Published:** 2022-09-20

**Authors:** Shuhei Nozaki, Ryuta Kinugasa, Katsutoshi Yaeshima, Takeshi Hashimoto, Masahiro Jinzaki, Naomichi Ogihara

**Affiliations:** 1 Laboratory of Human Evolutionary Biomechanics, Department of Biological Sciences, Graduate School of Science, The University of Tokyo, 7-3-1 Hongo, Bunkyo, Tokyo, 113-0033 Japan; 2 Department of Human Sciences, Kanagawa University, Kanagawa, 221-8686 Japan; 3 Sports Medicine Research Center, Keio University, Kanagawa, 223-8521 Japan; 4 Department of Radiology, Keio University School of Medicine, Tokyo, 160-8582 Japan

**Keywords:** Ultrasonography, Musculoskeletal system

## Abstract

This study aimed to identify the stiffness and natural length of the human plantar aponeurosis (PA) during quiet standing using ultrasound shear wave elastography. The shear wave velocity (SWV) of the PA in young healthy males and females (10 participants each) was measured by placing a probe in a hole in the floor plate. The change in the SWV with the passive dorsiflexion of the metatarsophalangeal (MP) joint was measured. The Young’s modulus of the PA was estimated to be 64.7 ± 9.4 kPa, which exponentially increased with MP joint dorsiflexion. The PA was estimated to have the natural length when the MP joint was plantarflexed by 13.8°, indicating that the PA is stretched by arch compression during standing. However, the present study demonstrated that the estimated stiffness for the natural length in quiet standing was significantly larger than that in the unloaded condition, revealing that the PA during standing is stiffened by elongation and through the possible activation of intrinsic muscles. Such quantitative information possibly contributes to the detailed biomechanical modeling of the human foot, facilitating an improved understanding of the mechanical functions and pathogenetic mechanisms of the PA during movements.

## Introduction

The human foot is known to be unique among primates in possessing a well-developed plantar aponeurosis (PA)^1–3^. The PA consists of mostly type 1 collagen fibers arranged in the longitudinal direction, spans the entire plantar side of the longitudinal arch of the foot, and is thought to be closely connected to the paratenon of the Achilles tendon throught the periosteum of the calcaneus^4,5^, allowing mechanical energy to be stored and released in each contact of the foot during walking and running^6–8^. In addition, the PA enables the foot to become a rigid lever during the push-off phase of walking and running by the so-called windlass mechanism. In particular, the PA is stretched to generate tension as the metatarsophalangeal (MP) joint is dorsiflexed in the late stance phase. This causes the stiffening of the midtarsal joint and entire foot during the push-off phase of walking and running for effective generation of propulsive forces^7,9,10^. For the detailed understanding of the biomechanics and motor control of the human foot, and to clarify the pathogenetic mechanism of foot disorders, it is important to accurately identify the biomechanical function of the PA in vivo during movements. To the best of our knowledge, the in vivo kinematic and kinetic PA properties during human movement has not been quantified due to the difficulty of capturing this information non-invasively.

Consequently, efforts have been made to computationally predict the kinematics and kinetics of the PA during walking and running using motion capture and biomechanical modeling^11–13^. For this, previous studies attempted to capture the mechanical behaviors of the human PA in vitro using a tensile testing machine^14–16^, while recent studies captured the mechanical behaviors of the PA in vivo using ultrasound elasticity imaging^17–29^. Based on the measured mechanical properties, the PA is modeled as a single or a bundle of linear springs^11,13,30,31^.

In these studies, the mechanical properties of the PA were measured in an unloaded condition (in the prone or supine position). However, soft tissues surrounding the PA may mechanically interact with the PA. This possibly affected the mechanical properties in a physiological loading condition, such as walking and running. For the accurate estimation of the PA tensile force, and other internal forces and moments generated in the foot using a foot model, the quantification of the mechanical properties of the PA in a physiological loading condition is necessary.

In this study, we aimed to identify the stiffness, linearity, and natural length of the PA spring in the human foot during a quiet standing condition by quantifying the change in the shear wave velocity (SWV) of the PA while passively increasing the dorsiflexion of the MP joint using ultrasound shear wave elastography. In addition, we investigated if there exist gender differences in the mechanical properties of the PA, because previous studies suggested that there were considerable sex differences in the stiffness of the PA^24^. Such quantitative information is expected to clarify the mechanical functions of the PA during movements and the pathogenetic mechanism of foot disorders.

## Methods

### Participants

Twenty healthy young participants (10 males and 10 females, age: 22.7 ± 3.0 years, height: 163.0 ± 8.2 cm, body mass: 58.9 ± 9.3 kg, and foot length: 23.8 ± 1.4 cm) were enrolled in this study. No participant had any history of any lower limb injuries or clinical symptoms, and did not exhibit any neurological disorders. This study was reviewed and approved by the Office for Life Science Research Ethics and Safety, the University of Tokyo, and it was performed according to the Declaration of Helsinki. Written informed consent was obtained from all the participants before data collection.

### Ultrasound imaging

An Aplio i700 ultrasound scanner (Canon Medical Systems Corporation, Otawara, Japan) with a linear array probe (effective length: 45 mm) was used to acquire the ultrasonic B-mode and shear wave images of the PA. The scanner was set at the musculoskeletal preset and high frequency (Res) mode. All measurements were performed by the same examiner, and the probe was manually operated.

**Figure 1:**
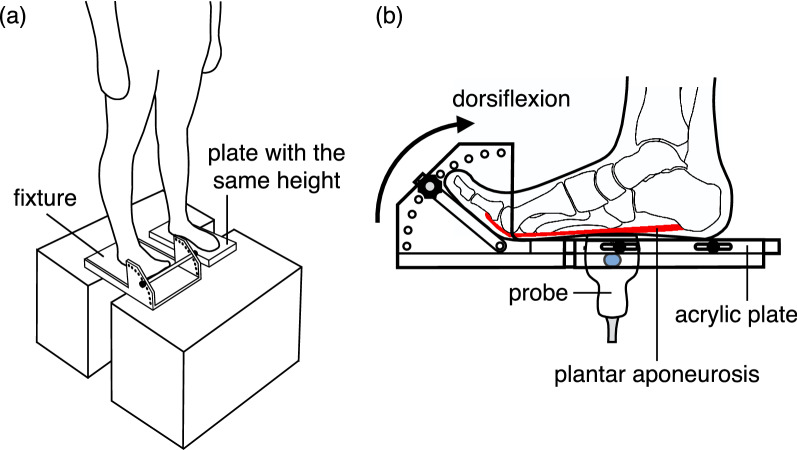
Schematic representation of the SWV measurement of the PA in the standing posture. The participants were requested to stand on a custom-made fixture (Takei Scientific Instruments, Niigata, Japan) with the measurement leg, and to place the other leg on a plate with the same height to maintain quiet standing posture. During standing, the MP joint was passively dorsiflexed at 0°, 10°, 20°, 30°, 40°, 50°, and 60° using the fixture (**b**). The longitudinal images of the middle site of the PA were scanned by placing the probe on the plantar surface of the foot at the navicular level through a circular hole (diameter = 65 mm) in the floor acrylic plate of the fixture that can slide along the anteroposterior direction to adjust the position of the hole with respect to the foot (**b**). *SWV* shear wave velocity, *PA* plantar aponeurosis, *MP* metatarsophalangeal.

To scan the middle site of the PA in the longitudinal direction, the probe was placed on the plantar surface of the foot parallel to the line connecting the calcaneus and the second metatarsal head (Fig. 1); the center of the probe was located at the level of the navicular tuberosity, as reported in a previous study^24^. During standing, the participants were instructed to equally distribute their weight on the feet in the mediolateral and anteroposterior directions, and to maintain their line of sight horizontal. Unloaded measurements were also conducted in the sitting posture with the leg horizontally placed on a platform, to ensure that no external forces were applied to the sole of the foot.

**Figure 2:**
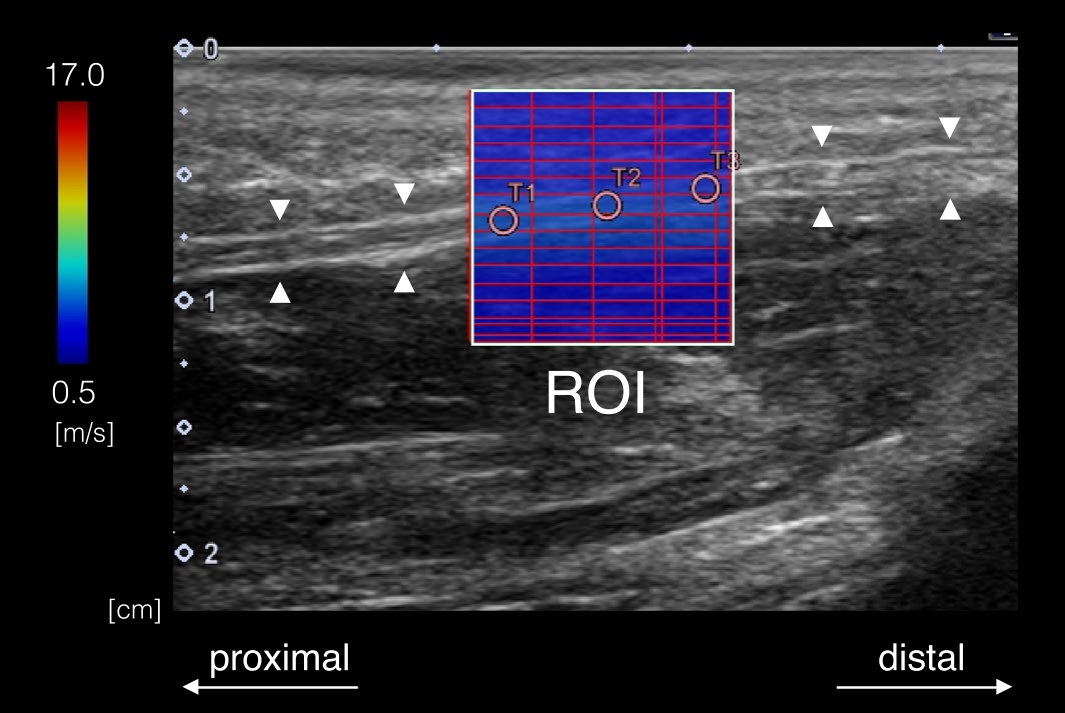
Representative shear wave image of the local SWV distribution of the aponeurosis in the square region of interest (RoI; 10 mm × 10 mm) (white grid). The PA is indicated by white triangles. The scale for the color code is provided to the left as SWV. SWV was calculated within three circles with a diameter of 1.25 mm equally positioned along the aponeurosis per one shear wave image.

One ultrasound B-mode image and five shear wave images of the local SWV distribution of the aponeurosis in the square region of interest (RoI; 10 mm × 10 mm) were obtained for each posture. The shear wave image was recorded as a color-coded map (Fig. 2). Thereafter, SWV was calculated within three circles with a diameter of 1.25 mm equally placed along the aponeurosis (Fig. 2) per one shear wave image using a measurement tool in the Aplio i700 prototype software (Canon Medical Systems Corporation, Otawara, Japan). A total of fifteen values of SWV were averaged to obtain the representative value for each posture. To evaluate the reproducibility of the SWV measurements, the precision was assessed for each participant by calculating a mean of the standard deviation of SWV.

To estimate the SWV and MP joint angle when the PA had natural length, SWV was plotted against different MP joint angles, and the following exponential function was fitted to the plots using the least-squares method:

1 $$\begin{array}{*{20}c} {V = \exp \left( {a\left( {\theta - b} \right)} \right) + c,} \\ \end{array}$$

where $V$ is the SWV; $\theta$ is the dorsiflexion angle of the MP joint; and $a$, $b,$ and $c$ are the coefficients determining the slope of the curve, dorsiflexion angle of the MP joint where the SWV steeply increases, and offset representing the SWV for the natural length of the PA, respectively. $a$, $b,$ and $c$ were obtained for each participant and the group mean by fitting the exponential function. Subsequently, the MP joint angle for the natural length of the PA was estimated. Since Eq. (1) is an asymptotical function, a threshold value of the SWV to define the onset of a rise of the asymptotic curve should be determined. Here we determined the threshold as 105% of the value $c$ because below this threshold the curve can be regarded completely horizontal. The MP joint angle for the natural length of the PA was therefore estimated by solving the following equation for $\theta_{0}$:

2 $$\begin{array}{*{20}c} {\exp \left( {a\left( {\theta_{0} - b} \right)} \right) = c \times 0.05,} \\ \end{array}$$

where $\theta_{0}$ is the MP joint angle for the natural length of the PA.

The Young’s modulus can be calculated based on SWV using the following equation^32,33^:

3 $$\begin{array}{*{20}c} {E = V^{2} \left( {2\rho \left( {1 + \nu } \right)} \right),} \\ \end{array}$$

where $E$ is the Young’s modulus, $V$ is the SWV, $\rho$ is the tissue density, and $\nu$ is the Poisson’s ratio. The tissue density and Poisson’s ratio were assumed to be 1000 kg/m^3^^34^ and 0.5^32,33^, respectively.

### Statistical analyses

The possible gender differences in the SWV for each condition and the coefficients of the exponential function (Eq. (1)) were investigated using *t*-tests or the Wilcoxon rank sum tests. The differences in the SWV among the unloaded and loaded (0°, 10°, 20°, 30°, 40°, 50°, and 60° of the MP joint dorsiflexion angle) conditions were analyzed using a one-way repeated measure ANOVA (within-subject factor: angle). If one-way ANOVA was significant, the post-hoc Tukey’s HSD test was performed to test if the SWV was influenced by the weight-bearing and MP joint angles. The statistical significance level was set at *P* < 0.05. Friedman test with a post-hoc Wilcoxon signed rank test for multiple comparisons, followed by Bonferroni correction with the adjusted *P*-value set at *P* < 0.005 (0.05/28), was used if the normality or homogeneity of variance was violated. The estimated SWV for the natural length of the PA was compared with the SWV in the unloaded condition using paired *t*-test or Wilcoxon signed rank test. Data processing and analyses were implemented in R software, version 3.5.2^35^.

## Results

**Figure 3:**
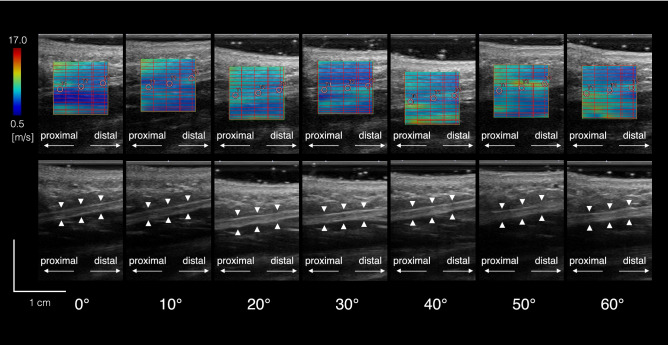
Representative ultrasound shear wave and B-mode images of the PA (upper and lower rows, respectively) that were obtained at 0°, 10°, 20°, 30°, 40°, 50°, and 60° of MP joint dorsiflexion under the weight-bearing condition. The PA is indicated by white triangles. The scale for the color code is provided to the left as SWV.

**Figure 4:**
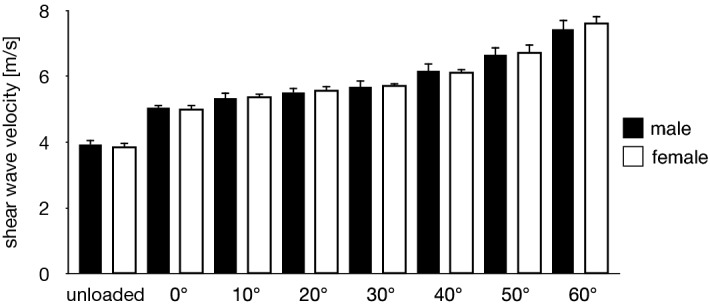
Gender-based comparisons of the SWV of the PA under all conditions. The error bars indicate standard deviations. There were no significant gender-associated differences in the SWV of the PA under all conditions.

**Figure 5:**
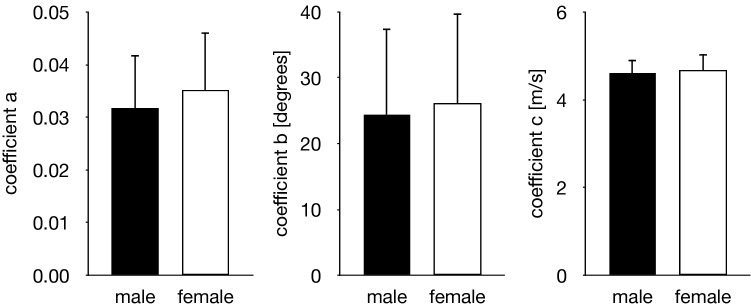
Gender-based comparisons of the three coefficients of the exponential function. The error bars indicate standard deviations. There were no gender-associated significant differences in the three coefficients of the exponential function.

There were no significant gender-associated differences in the SWV of the PA (Fig. 3) in all conditions (Fig. 4) and in the three coefficients of the exponential function (Fig. 5). Therefore, the data of the SWV and the coefficients of the exponential function in females and males were pooled in the following analyses.

**Figure 6:**
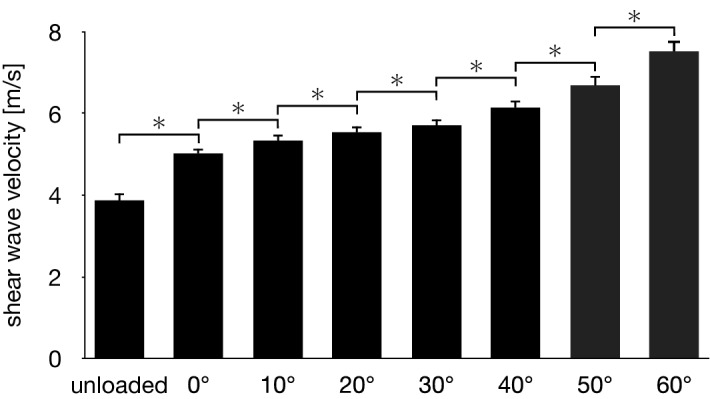
Comparisons between the SWV of the PA under all conditions. The error bars indicate standard deviations. The asterisk indicates that there was a significant difference in the SWV of the PA among the MP joint angles.

**Figure 7:**
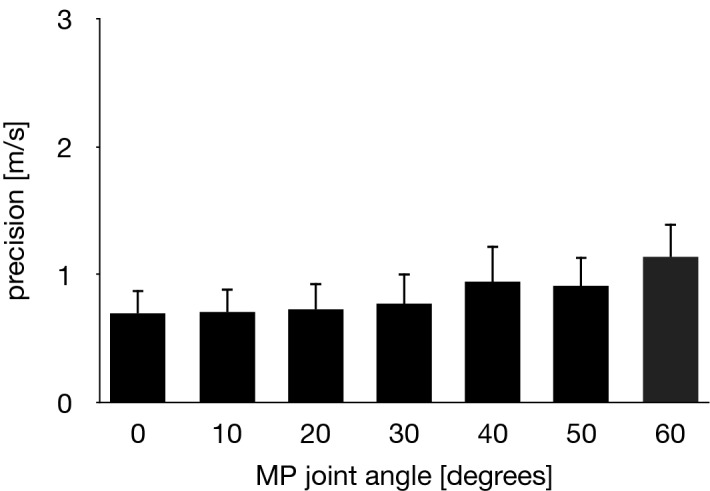
Precision of the SWV measurements of the PA. Error bars indicate standard deviation.

**Figure 8:**
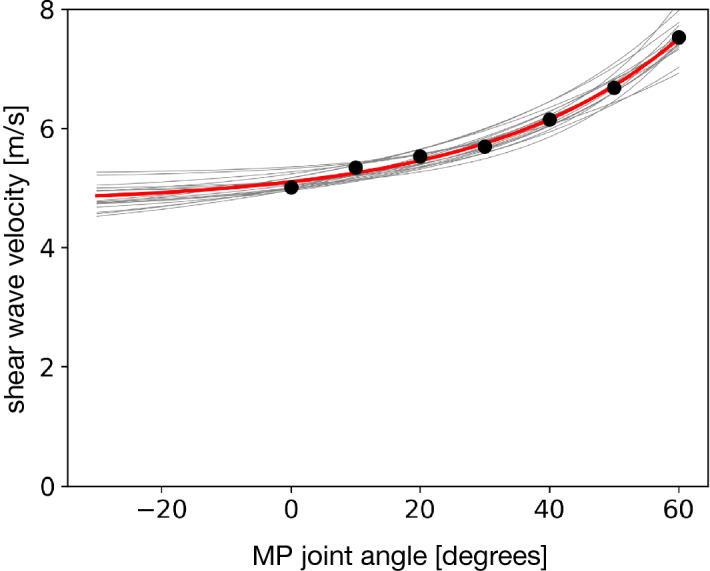
Exponential curve (red curve) fitted to the group mean SWV plots (black plots) against the different MP joint dorsiflexion angles and the exponential curves of the 20 individual participants (gray curves).

**Figure 9:**
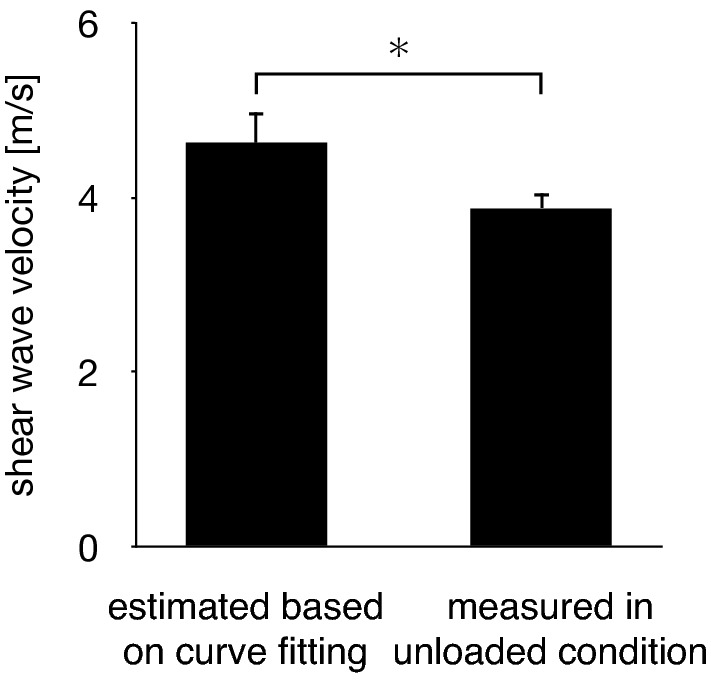
Comparison between the estimated SWV for the natural length of the PA (value of $c$) and the SWV in the unloaded condition. The error bars indicate standard deviations. The asterisk indicates that there was a significant difference.

The SWV of the PA increased with the passively increasing dorsiflexion angle of the MP joint (Fig. 6). Friedman test exhibited significant differences in the SWV of the PA among the unloaded and loaded (0°, 10°, 20°, 30°, 40°, 50° and 60° of the MP joint dorsiflexion angle) conditions (*x*^2^ = 140, *P* < 0.0001). The SWV of the PA significantly increased as the MP joint was dorsiflexed by 10° (*P* < 0.0001) (Fig. 6). The precision of the SWV measurement was < ~ 1 m/s, which gradually increased with increasing dorsiflexion angle of the MP joint (Fig. 7), possibly due to the fact that the amplitude of excited shear wave hence the signal-to-noise ratio decrease when the wave propagates stretched, stiffer tissues. The SWV exponentially increased as the MP joint was dorsiflexed in the loaded condition (Fig. 8). The mean coefficients, *a*, *b*, and *c*, in the fitted exponential curve were 0.0334 ± 0.0101, 25.2 ± 13.1, and 4.63 ± 0.34, respectively. The Young’s modulus of the PA was estimated to be 64.7 ± 9.4 kPa for the natural length of the PA. The PA was estimated to exhibit its natural length when the MP joint was plantarflexed by 13.8°. Furthermore, the estimated SWV for the natural length of the PA (value of $c$) was significantly larger than that under the unloaded condition (*P* < 0.0001, Fig. 9).

## Discussion

In this study, we quantified the stiffness of the human PA under a physiological loading condition. Such information on the mechanical properties of the PA contributes to the detailed biomechanical modeling of the human foot, leading to an improved understanding of the mechanical functions of the PA during movements, such as walking, running, and jump landing, and of the pathogenesis of the plantar fasciitis and plantar fibromatosis^23,36^.

Our value of PA Young’s modulus (SWV = 4.6 m/s) was lower than previously reported (SWV = 5.1–8.6 m/s)^17–21,23–29^. Although the exact cause of this discrepancy remains unclear, it might be attributable to the difference in the leg posture during measurement. In previous studies, SWV measurements were performed when participants were in a supine or prone position on a bed, and therefore, the knee was completely extended. This complete extension of the knee might have stretched the gastrocnemius muscle^37^ and generated passive tensile force to pull up the calcaneal tuberosity, possibly leading to the stretching and stiffening of the PA even though the foot was unloaded. In contrast, the present study measured SWV when the knee was approximately 90° flexed in the unloading condition. Even in the standing posture, the knee was slightly flexed; it was not completely extended during measurements. Therefore, differences in the leg posture, particularly the knee joint angle, might considerably influence the SWV measurements. This possibility must be further investigated for an accurate and more consistent quantification of the mechanical properties of the PA.

The SWV exponentially increased with the dorsiflexion of the MP joint (Fig. 8), indicating that the PA is not a linear spring, which is in agreement with the previous cadaveric^14–16^ and ultrasound elasticity imaging studies^26^. In the construction of the musculoskeletal models of the human foot incorporating the PA, the PA is often modeled as a single or a bundle of linear springs^11,13,30,31^. However, both the current and previous studies demonstrated the mechanical property of the PA to be not linear elastic. Therefore, caution is required when computationally predicting the kinetics of the PA during movements using a foot musculoskeletal model.

The current study estimated that the MP joint was plantarflexed by 13.8° for the natural length of the PA. Assuming the radius of the metatarsal head to be ca. 9 mm^11^, the plantarflexion angle of 13.8° approximately corresponds to a 2 mm change in the PA length. This is consistent with a recent cadaver study that showed that the medial arch was elongated by approximately 2 mm when the foot was axially loaded by approximately half body weight^38^. Therefore, the natural length of the PA can be approximately estimated as the length of PA during quiet standing (when MP angle = 0°) subtracted by 2 mm.

However, the estimated SWV for the natural length of the PA is significantly larger than that in the unloaded condition (Fig. 9), although these values ought to be equal if the stiffness of the PA changes by only the elongation of the PA. This contradiction might be because the stiffness of the PA during quiet standing is determined by not only the change in PA length but also the activation of the intrinsic foot muscles attached to the PA. For example, the inner side of the PA is firmly attached to the superficial intrinsic foot muscles, such as abductor hallucis, flexor digitorum brevis, and abductor digiti minimi^5^. An electromyographic study has demonstrated that the abductor hallucis and flexor digitorum brevis were activated in the standing posture^39^. Therefore, the activation of the intrinsic foot muscles might increase the stiffness of the PA during quiet standing, in addition to the elongation of the PA. During human walking, the intrinsic foot muscles reportedly contribute to the efficient generation of the propulsion force in the late stance phase^40^. Our results suggested that the interaction between the stiffness of PA and the activation of the intrinsic muscles of the foot should be considered for the accurate modeling of the mechanics of the PA during locomotion.

It has been previously reported that there were considerable gender differences in the stiffness of the PA^24^. However, in the current study, no significant gender-associated differences in the SWV of the PA were found in any condition. This is in agreement with Taş (2018)^41^—in that the SWV of the PA does not differ between males and females—but in disagreement with Shiotani et al. (2019)^24^—in that the SWV of the PA in females is significantly larger than that in males. However, tendinous connective tissues are usually considered mechanically weaker in females than in males^42^; the Achilles^43–45^ and patellar^46,47^ tendons are reportedly softer in females than in males, although some studies did not observe such gender-based differences^48–53^. Therefore, we believe that the stiffness is not larger in females than in males, and there exist no gender-associated differences in the SWV of the PA; however, this must be confirmed through further studies.

The present study has some limitations. First, the number of the participants were relatively small, and the only young participants (range 19–33 years) were enrolled. Although we believe that the present study successively quantified the mechanical properties of the PA during quiet standing, they should be investigated with larger sample size and more wide range of ages in future study. Second, although the present study investigated the increase in the PA stiffness during the passive dorsiflexion of the MP joint during quiet standing, this experimental condition was certainly not identical to that of the foot at the time of toe off during walking. To directly infer the mechanical characteristics of the PA during the functioning of the windlass mechanism in vivo, further technical innovations are certainly necessary to directly capture the PA material property without using a probe, as placing a probe on the plantar surface of the foot certainly renders normal gait impossible.

In conclusion, the Young’s modulus of the PA was estimated to be 64.7 ± 9.4 kPa during quiet standing and 45.30 ± 3.68 kPa in the unloaded condition, which exponentially increased with the dorsiflexion of the MP joint, suggesting that the PA was a nonlinear spring. The PA was estimated to have natural length when the MP joint was plantarflexed by 13.8°, indicating that the PA was elongated by approximately 2 mm during quiet standing owing to the compression of longitudinal arch. Furthermore, our study showed that the estimated SWV for the natural length of the PA was significantly larger than that in the unloaded condition, suggesting that the SWV of the PA during quiet standing was stiffened by elongation and through the possible activation of the intrinsic foot muscles located along and possibly attached to the PA. Such quantitative information possibly contributes to the detailed biomechanical modeling of the human foot, facilitating an improved understanding of the mechanical functions and pathogenetic mechanisms of the PA during movements.

## Data Availability

The data supporting the findings of this study are available from the corresponding authors upon reasonable request.
